# Risk of wheezing and asthma exacerbation in children treated with paracetamol versus ibuprofen: a systematic review and meta-analysis of randomised controlled trials

**DOI:** 10.1186/s12890-020-1102-5

**Published:** 2020-03-23

**Authors:** Mohamed Sherbash, Luis Furuya-Kanamori, Joanne Daghfal Nader, Lukman Thalib

**Affiliations:** 1 Hamad Medical Corporation, Doha, Qatar; 2 Research School of Population Health, Australian National University, Acton, ACT Australia; 3 Department of Public Health, College of Health Sciences, QU Health, Qatar University, Doha, Qatar

**Keywords:** Paracetamol, Acetaminophen, Ibuprofen, Wheezing, Asthma, Meta-analysis

## Abstract

**Background:**

Paracetamol and ibuprofen are the most commonly used medications for fever and pain management in children. While the efficacy appears similar with both drugs, there are contradictory findings related to adverse events. In particular, incidence of wheezing and asthma among children taking paracetamol compared to ibuprofen, remain unsettled.

**Methods:**

We conducted a meta-analysis of randomized controlled trials (RCTs) that compared wheezing and asthma exacerbations in children taking paracetamol versus ibuprofen. A comprehensive search was conducted in five databases. RCTs reporting on cases of wheezing or asthma exacerbations in infants or children after the administration of paracetamol or ibuprofen were included. The pooled effect size was estimated using the Peto’s odds ratio.

**Results:**

Five RCTs with 85,095 children were included in the analysis. The pooled estimate (OR 1.05; 95%CI 0.76–1.46) revealed no difference in the odds of developing asthma or presenting an exacerbation of asthma in children who received paracetamol compared to ibuprofen. When the analysis was restricted to RCTs that examined the incidence of asthma exacerbation or wheezing, the pooled estimate remained similar (OR 1.01; 95%CI 0.63–1.64). Additional bias adjusted quality effect sensitivity model yielded similar results (RR 1.03; 95%CI 0.84–1.28).

**Conclusion:**

Although, Ibuprofen and paracetamol appear to have similar tolerance and safety profiles in terms of incidence of asthma exacerbations in children, we suggest high quality trials with clear definition of asthma outcomes after receiving ibuprofen or paracetamol at varying doses with longer follow-up are warranted for any conclusive finding.

## Background

Paracetamol, also known as acetaminophen, is one of most commonly used over-the-counter medication to treat pain and fever. Paracetamol is perceived as a safe medication to control pain and fever in children, yet evidence from multiple epidemiological studies have shown a potential association between its use and asthma. Epidemiological studies have found an increased risk across difference populations as well as a dose-response relationship [1–8]. A meta-analysis found the odds of wheezing doubles (OR 1.97; 95%CI 1.51–2.56) in children exposed to paracetamol [9]. On the other hand, a recent study by Walsh and Rothenberg found a decreased in risk of wheezing in children when exposed to paracetamol for an episode of bronchiolitis or respiratory tract infection [10].

The increasing evidence accumulated over the past 30 years about the risk of asthma exacerbations associated with paracetamol has led clinicians to recommend a change in practice, particularly in children [11–13]. Among the alternatives to treat pain and fever in children, one that stands out is ibuprofen. A meta-analysis by Pierce and Voss found that ibuprofen is more effective in reducing pain and fever than paracetamol in paediatric population [14]. They also reported that ibuprofen and paracetamol had similar rates of adverse events; however, they did not assess the risk of asthma [14]. Ibuprofen as a nonsteroidal anti-inflammatory drug (NSAID), inhibits cyclooxygenase (COX) and reduces prostaglandin synthesis. That in turn causes reduction in fever, inflammation, and pain. However, the inhibition of the COX pathway can also activate the lipoxygenase pathway, leading to an increase in leukotriene synthesis, and resulting in bronchospasms and asthma exacerbation [15].

The aim of this meta-analysis is to quantify the risk of wheezing and asthma exacerbations in paediatric population taking ibuprofen and paracetamol. Our findings are expected to help guide the paediatricians in management of fever and pain and drug choices.

## Methods

Findings from this systematic review and meta-analysis are presented following the recommendations from the Preferred Reporting Items for Systematic Reviews and Meta-Analyses (PRISMA). The review was registered with the International Prospective Register of Systematic Reviews (CRD42017080165).

### Search strategy and study selection

The search strategy was conducted in PubMed, Science Citation Index, Embase, Cochrane library, and ClinicalTrials.gov. The following search terms were included (paracetamol OR tylenol OR acetaminophen) AND ibuprofen AND (pediatrics OR children OR infants). All databases were searched from their inception until September 2017. The search was updated in July 2019 and was supplemented with a PubMed similarity search as well as a forwards and backwards citation search for the articles included in the meta-analysis. The references lists from relevant systematic reviews and meta-analyses were hand searched.

Eligible studies were RCTs conducted in humans that included paracetamol and ibuprofen within their study arms. To be considered for inclusion, the RCTs had to include children (≤12 years) and reported the number of cases of wheezing, asthma and/or asthma exacerbations. The outcome of interest (i.e.wheezing, asthma or asthma exacerbation) could be reported either as the primary or secondary outcome or as an adverse event in the RCT. Studies were excluded if the study arms included co-interventions (e.g. paracetamol plus codeine). No language restriction was applied. Two authors (MS and JN) independently screened the articles. Disagreement was resolved through consensus.

### Data extraction and quality assessment

A spreadsheet was used for data extraction. The following items were extracted by one of the authors (MS) author, year of publication, study setting and characteristics of the study population, follow-up time, characteristics of the interventions (e.g. dose of medication), sample size, and number of cases of wheezing or asthma excerbations. The extracted data was cross-checked by a second author (JD). The same two authors assessed the risk of bias of the RCTs using the Cochrane collaboration’s tool for assessing the bias in randomized trials [16].

### Statistical analysis

The effect measure of interest was the odds ratio (OR) of developing asthma exacerbations or wheezing after exposure to paracetamol compared to ibuprofen. The pooled OR was estimated using the Peto’s method as the outcome was rare (three arms contained zero events). Statistical heterogeneity among studies was assessed using the *I*^2^, an *I*^2^ > 50% was considered substantial between-study heterogeneity. Sensitivity analyses were conducted by excluding studies that measured exacerbation of asthma as their outcome as well as including the studies’ quality information (i.e. risk of bias) into the model through the quality effects (QE) model [17]. Publication bias was investigated using the Doi plot and LFK index [18]. The analysis was conducted in MetaXL version 5.3 (EpiGear Int Pty Ltd.; Sunrise Beach; Australia; http://www.epigear.com).

## Results

### Selection of studies

**Fig. 1:**
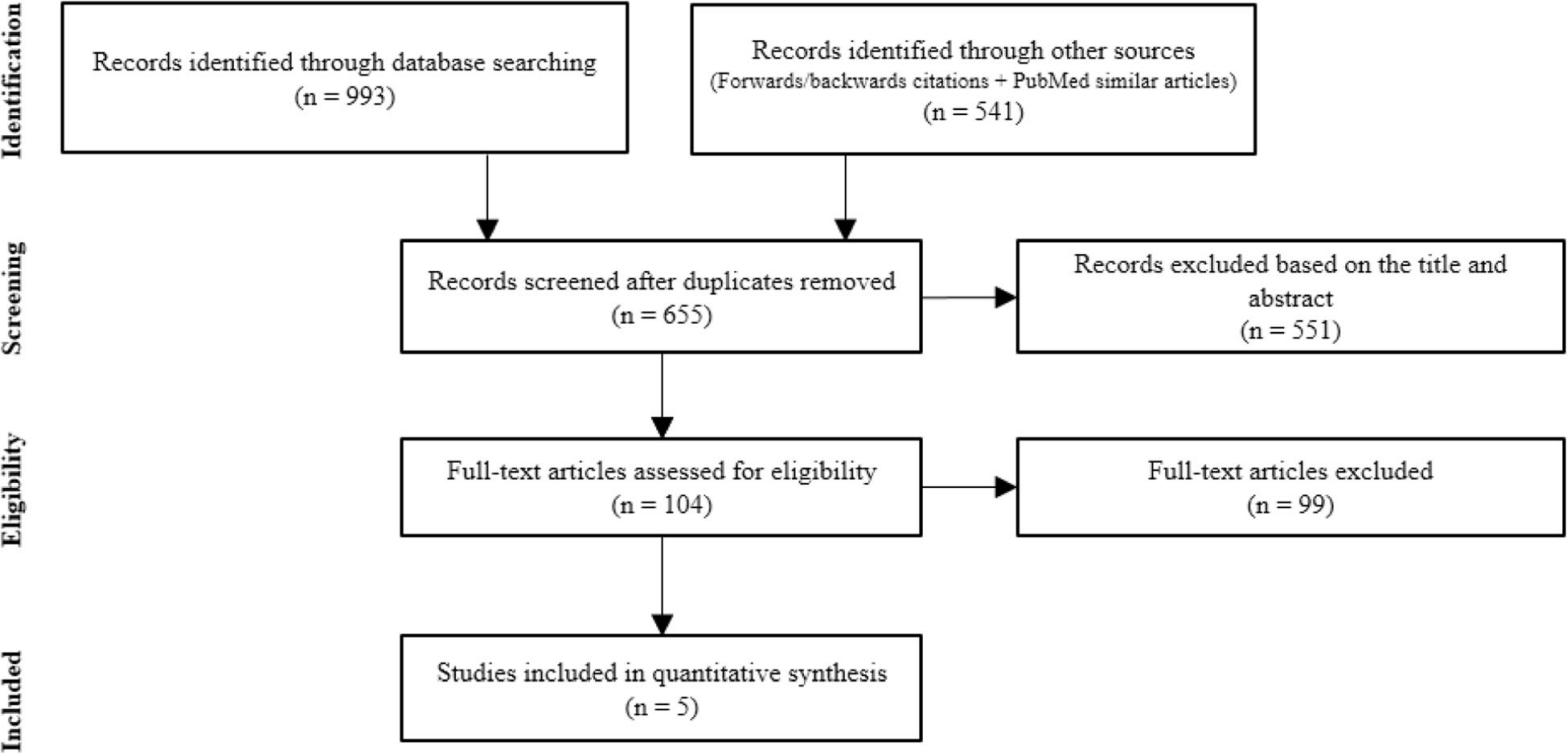
PRISMA flow diagram

The search identified 655 publications, 104 articles remained after title and abstract screening and the full text of the articles were examined to assess their eligibility. Eight publications met the inclusion criteria. Four publications [8, 19–21] used subsets of the Boston University Fever Study data, only the publication with larger sample size [19] was included in the meta-analysis. Therefore, five RCTs with 85,095 children were included in the meta-analysis (Fig. 1).

### Characteristics of included studies

Four RCTs were conducted in hosptails [22–25], while one was conducted in paediatric and family medicine practices [19]. The age of the participants ranged from 2 months to 12 years with a mean age between 21 and 40 months. Four RCTs [19, 22, 23, 25] included only febrile children, their outcome of interest was episodes of asthma excerbations after the administration of paracetamol or ibuprofen, and had a relative short follow-up period ranging from 6 h to 28 days. Sheehan et al. [24] included afebrile children with asthma, thus the outcome of interest was exacerbation of asthma or wheezing after receiving paracetamol or ibuprofen, and had a longer follow-up period of 48 weeks (Table 1). Overall, the risk of bias was low among the RCTs, the most common deficiency was blinding of outcome assessment, followed by allocation concealment and blinding of participants and personnel (Table 2).

**Table 1:** Characteristics of the included randomised controlled trials

Author, year	Setting	Study population	Proportion of females (%)	Mean/median age (months)	Follow-up duration (days)	Dose of paracetamol/ibuprofen	Number of participants paracetamol/ ibuprofen	Cases of asthma paracetamol/ ibuprofen
Lesko et al. 1995 [19]	Outpatient paediatric and family medicine practice throughout the US (1991–1993)	Children aged 6 months to 12 years with acute febrile illness (Boston University Fever Study)	48	40	28	12 mg/kg / 5-10 mg/kg	28,130 / 55,739	24 / 44
Luo et al. 2017 [22]	Tertiary hospital in China (2013–2014)	Febrile children aged 6 months to 5 years attending the emergency room	40	30	5	10 mg/kg / 10 mg/kg	158 / 157	0 / 2
McIntyre and Hull 1996 [23]	Tertiary hospital in the UK	Febrile children aged 2 months to 12 years	41	21	3	50 mg/kg / 20 mg/kg	74 / 76	2 / 0
Sheehan et al. 2016 [24]	18 hospitals in the US (2013–2015)	Children aged 1 to 6 years receiving long-term step 2 asthma-controller therapy (The Acetaminophen versus Ibuprofen in Children with Asthma trial)	40	40	336	15 mg/kg / 9.4 mg/kg	150 / 150	74 / 70^a^
Wong et al. 2001 [25]	University hospitals in Argentina, Mexico, Chile, and Brazil (1998)	Febrile children aged 6 months to 6 years	46	30	0.25	12 mg/kg / 5-10 mg/kg	210 / 209	0 / 2

^a^Cases of asthma exacerbations

**Table 2:** Assessment of risk of bias of the included randomised controlled trials

Author, year	Random sequence generation	Allocation concealment	Blinding of participants and personnel	Blinding of outcome assessment	Incomplete outcome data	Selective reporting	Exposure assessment^a^	Outcome assessment^a^
Lesko et al. 1995 [19]	Low risk	Low risk	Low risk	Low risk	Low risk	Low risk	High risk	High risk
Luo et al. 2017 [22]	Low risk	Low risk	High risk	High risk	Low risk	Low risk	Low risk	Low risk
McIntyre and Hull 1996 [23]	Low risk	High risk	High risk	High risk	Low risk	Low risk	Low risk	Low risk
Sheehan et al. 2016 [24]	Low risk	Low risk	Low risk	Low risk	High risk	Low risk	Unclear	Low risk
Wong et al. 2001 [25]	Low risk	High risk	Low risk	High risk	Low risk	Low risk	Low risk	Low risk

^a^ Exposure and outcome assessment were considered low risk if the information was obtained from medical records or directly recorded by the investigators, and high risk if it was self-reported by the parents or guardians of the children

The other items of the risk of bias were classified as low or high risk following the Cochrane collaboration’s tool for assessing the bias in randomized trials [16]

### Quantitative analysis

**Fig. 2:**
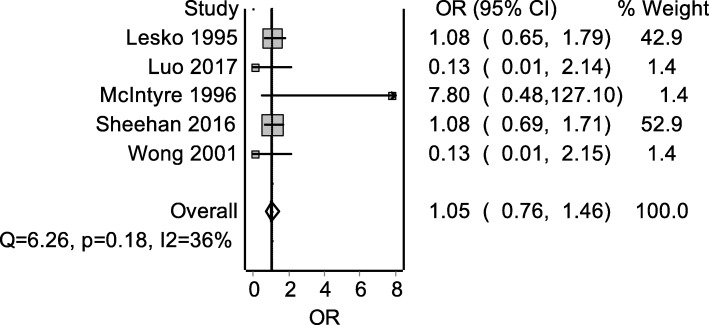
Forest plot of the Peto’s odds ratio of asthma in children who received paracetamol compared to ibuprofen

**Fig. 3:**
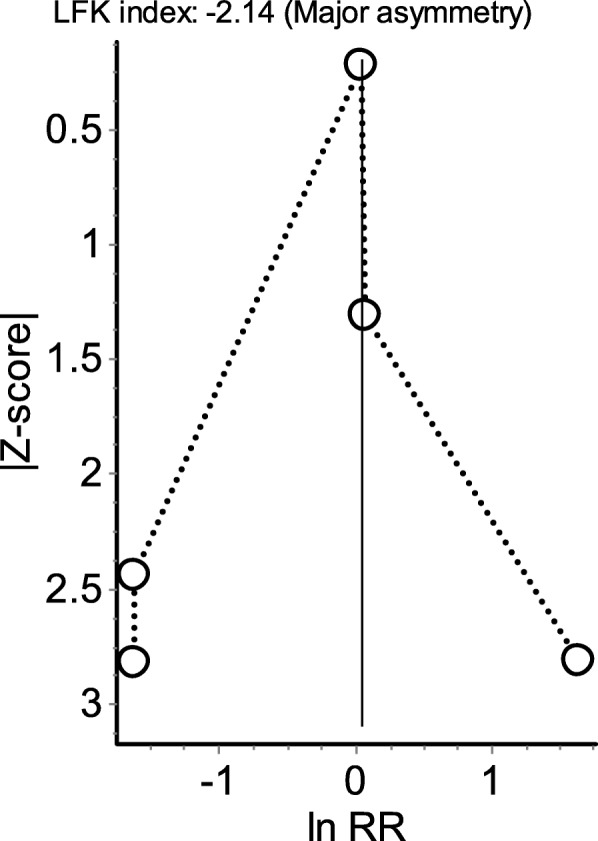
Doi plot and LFK index for the detection of publication bias

The point estimates of two RCTs indicated lower risk of asthma exacerbations with paracetamol [22, 25], two RCTs point estimates indicated no difference [19, 24], while the last RCT point estimate favoured the use of ibuprofen [23]. Moderate statistical heterogeneity (*I*^2^ = 36%) among the five RCTs was observed. The pooled estimate (OR 1.05; 95%CI 0.76–1.46) revealed that there is no difference in the odds of developing asthma exacerbations or wheezing in children who received paracetamol compared to ibuprofen (Fig. 2). When the analysis was restricted to RCTs that examined the episodes of asthma or exacerbations of asthma as their outcome of interest, the pooled estimate did not change (OR 1.01; 95%CI 0.63–1.64). The QE model using the additional information from the quality assessment of each studies yielded similar results (RR 1.03; 95%CI 0.84–1.28). The Doi plot and the LFK index (− 2.14) indicated major asymmetry in favour of RCTs reporting less episodes of asthma exacerbations with paracetamol compared to ibuprofen (Fig. 3).

## Discussion

Although efficacy, safety, and tolerability of ibuprofen and paracetamol in children have been investigated [14, 26]; only few studies have investigated the association between asthma and the use of these medications. After a comprehensive search, only five relevant RCTs were identified and included in this review, which points out to the paucity of data in this area of research. The pooled estimate indicates that there is no statistical nor clinically important differences between the use of ibuprofen or paracetamol in the paediatric population in relation to asthma morbidity.

These null findings appear to be similar even among very young children. Lesko and Mitchell used a subset of their original data and compared the safety profile of these medications in children less than 2 years [21]. They found that the risk of hospital admissions due to asthma/bronchiolitis was similar with ibuprofen and paracetamol. Such a negative findings support the theory that risk of asthma morbidity is similar with ibuprofen and paracetamol. Their study also showed that the effect is not modified by the age. This is particular pertinent as the sample size in their study (*n* = 27,065) was large with sufficient power to detect even a small differences, if existed between drugs.

Potential biological mechanisms that underpin the role of these drugs in asthma exacerbations have been postulated. Paracetamol is related to impaired lung function and asthma through a number of possible mechanisms; first being through oxidative stress. As paracetamol can lead to reduction of antioxidant glutathione concentration in the lungs, that can increase the oxidative stress in asthmatic patients. Bronchospasm through leukotriene release can be triggered by reactive oxygen species, which can greatly affect sensitive asthmatic patients [27, 28]. A second mechanism is related to the lack of COX-2 inhibition by paracetamol which can trigger immune mediated response through T helper cells which increase sensitivity to an allergic tendency to different stimuli. Another study suggested that the frequent use of paracetamol can result in consistent decrease in intracellular glutathione levels which can be linked to asthma morbidity [29, 30]. On the other hand, ibuprofen were thought to induce asthma symptoms through COX-1 enzyme blockage which increases thromboxane production and decreases production of prostaglandins that have anti-inflammatory effect, leading to an increased release of pro-inflammatory leukotrienes which are chemical triggers for severe asthma exacerbations and allergy-like symptoms [31]. Although, the plausible biological pathways for paracetamol and ibuprofen to produce bronchospasms are different, findings from our study revealed that the net effect in bronchospasms is similar between both medications.

Our findings should be understood in the light of a number of limitations. The follow-up duration in four out of the five RCTs included in the meta-analysis was ≤28 days. The association between medication exposure and diagnosis of asthma may require a longer follow-up period as symptoms could develop after 4 weeks. A large RCT, PIPPA Tamariki (https://pippatamariki.ac.nz) with a proposed follow-up of 6 year will provide more evidence about the risk of developing asthma in children exposed paracetamol compared to children exposed to ibuprofen within their first year of life. Asthma was not the primary or secondary outcome and was reported as an adverse event in four RCTs. The severity of morbidity from asthma was not consistent across all studies as one RCT included “mild wheezing” [23], while another used asthma exacerbation that required systemic glucocorticoids as the outcome of interest [24]. There were also variation in the dose of the interventions between RCTs as well as in the follow up time that ranged from 6 h to 48 weeks.

## Conclusions

Our finding of similar tolerance and safety profiles of paracetamol and ibuprofen in terms of incidence or exacerbation of asthma in children is based on five RCTs. As there were paucity of research in this area, high quality trials with clear definition of asthma outcomes after receiving ibuprofen or paracetamol at varying doses with longer follow-up are warranted.

## Data Availability

Data sharing not applicable to this article as the data used in the current study was extracted from published studies.

## Abbreviations

CI: Confidence interval
COX: Cyclooxygenase
IVhet: Inverse variance heterogeneity
LFK: Luis Furuya-Kanamori
NSAID: Nonsteroidal anti-inflammatory drug
OR: Odds ratio
PRISMA: Preferred Reporting Items for Systematic Reviews and Meta-Analyses.
QE: Quality effects
RCT: Randomised control trial
RR: Relative risk
